# A study on the effects of stress and hopelessness in isolated COVID-19 patients in relation to severity of infection

**DOI:** 10.1192/j.eurpsy.2021.1766

**Published:** 2021-08-13

**Authors:** S. Mittal, S. Nagendran

**Affiliations:** Psychiatry, Teerthanker mahaveer medical College and research centre, Moradabad, India

**Keywords:** Covid-19 severity, stress, coronavirus, Hopelessness

## Abstract

**Introduction:**

In India, Coronavirus pandemic started in the month of march 2020 and is growing day by day. In view of India being one of the most populous countries, it is hard to follow social distancing and abide by the lockdown rules. Therefore, as of December 2020, total number of covid-19 cases has crossed the 10 million. But the recovery rate in India is high, so the fear due to Covid-19 has decreased in intensity.

**Objectives:**

To assess level of perceived stress in isolated covid-19 patients To assess level of hopelessness in isolated covid-19 patients

**Methods:**

30 Patients of diagnosed Covid-19 positive,who were isolated in covid care setting in Uttar Pradesh(India),above 18yrs of age,of both sexes and willing to participate in the study were included, their socio-demographic data collected. Beck’s hopelessness scale and Perceived stress scale were administered. Infection severity upto moderate was selected and ICU patients were excluded. Results were analysed using SPSS software.

**Results:**

It was observed that level of hopelessness increased with increasing age and increasing severity of covid-19.Level of perceived stress also increased with increasing age and increasing covid severity. There was no relation seen between hopelessness level and perceived stress level and no difference was seen in the levels of hopelessness and perceived stress between the two sexes.

**Conclusions:**

Levels of hopelessness and stress increased with increasing age and increasing severity of covid-19.No relation seen between hopelessness level and perceived stress level and no difference was seen in the levels of hopelessness and perceived stress between the two sexes.

**Disclosure:**

No significant relationships.

